# Personal

**Published:** 1903-02

**Authors:** 


### PERSONAL.

Dr. William C. Krauss, of Buffalo, announces his removal from 371 to 479 Delaware Avenue. Hours: 8-11; 1-4 and by appointment. Telephone, Tupper 96.

---

Dr. Frederick H. Millener, of Buffalo, who has been ill for some weeks, has recovered his health and resumed his professional practice, which is limited to diseases of the ear, nose and throat.

---

Dr. D. J. Constantine, of Buffalo, who for several years past has been an inspector in the department of health, has resigned that office and will hereafter devote himself entirely to the practice of medicine which now requires his whole time.

---

Dr. Adolph H. Urban, of Buffalo, has been appointed clinical instructor of diseases of nose, throat and ear at the Buffalo University, Medical Department, also otologist to the German Hospital Dispensary. His office and residence are at 523 Franklin Street. Hours: 9 to 12 a.m., 5 to 6 p.m. and by appointment. Telephone Tupper, 1436.

---

Dr. Willis G. Gregory, of Buffalo, was elected first vice-president of the New York State Board of Pharmacy, at its recent annual meeting, held at Albany. Dr. Gregory has been a member of the board for some years.

---

Dr. Eva V. Mead, of Buffalo, has established her office at 426 Dearborn Street. Hours: 4 to 5 and 7 to 8 p.m. Telephone, North 115.

---

Dr. Beatrice Todd Hagen, U. of B., 1900, has removed from 407 Fargo Avenue, to The Netherland, 267 Franklin Street. Hours: 9-10 a.m.; 2-4 p.m.

---

Dr. R. B. Granger, of New York, for many years managing editor of the *New York Medical Journal*, announces to his medical friends and acquaintances that he has become associated with the medical publishing house of W. B. Saunders & Company, of Philadelphia, as its representative in New York City. A fine suite of offices has been opened in the Fuller Building, at the junction of Fifth Avenue, Twenty-third Street, and Broadway, where he will be pleased to receive his friends. Dr. Granger's acquaintance with the American profession is very large and all will be interested in learning of his success in his new relations, though he will be missed from the position which he has honorably filled for so many years.

